# Optimization of Water and N Regulation for Mung Bean (*Vigna radiata* L.) Cultivation Under Drip Irrigation Using TOPSIS Method in Mollisols Region of Northeast China

**DOI:** 10.3390/plants15040669

**Published:** 2026-02-23

**Authors:** Dehao Lu, Ying Liu, Yimeng Zhu, Lili Jiang, Tianyi Wang, Peng Chen, Tangzhe Nie, Xingtao Xiao

**Affiliations:** 1 School of Water Conservancy and Electric Power, Heilongjiang University, Harbin 150080, China; 2212006@s.hlju.edu.cn (D.L.); liuying@hlju.edu.cn (Y.L.); zhuyimeng61@163.com (Y.Z.); 2023086@hlju.edu.cn (L.J.); 2025069@hlju.edu.cn (T.W.); 2 College of Agricultural Science and Engineering, Hohai University, Nanjing 211100, China; chenpeng_isotope@163.com; 3 Heilongjiang Provincial Hydrology and Water Resources Center, Harbin 150001, China

**Keywords:** drip irrigation, N partial factor productivity, TOPSIS, *Vigna radiata* L., water and N regulation, water use efficiency

## Abstract

Optimizing the coupling effect between irrigation and N fertilizer to balance mung bean (*Vigna radiata* L.) production and the effective utilization of water and fertilizer resources is an important challenge for sustainable agricultural production. In this study, a field drip irrigation experiment was conducted on Mollisols in Northeast China, and twelve treatments were performed: four levels of soil water content (W1, 80~100% of field capacity; W2, 70~90% of field capacity; W3, 60~80% of field capacity; W4, rainfed condition) and three N application treatments (40 (N1), 80 (N2), and 120 (N3) kg/ha). We analyzed the coupling effects of water and N levels on mung bean growth, yield and yield components, water consumption, water use efficiency (WUE) and N partial factor productivity (PFP) in 2021 and 2022 and screened the optimal water and N regulation by the TOPSIS method. The results showed that the amount of N application dominated the regulation of water and N. In the first year, plant height, stem diameter, number of seeds per pod, 100-seeds weight, yield, aboveground dry matter accumulation, WUE, and PFP in mung bean decreased with increasing N applications at the same irrigation treatment. Furthermore, except for WUE, all results of the W3N1 treatment reached the highest levels, at 79.14 cm, 13 mm, 12.4, 6.2 g, 1430.45 kg/ha, 79.27 g (the drumming stage), and 35.76 kg/kg, respectively. The second year, plant height, stem diameter, yield and WUE had an increasing trend with increasing N applications at the W1. Based on the TOPSIS method, the W3N1 treatment could obtain the optimal comprehensive benefits of yield, WUE and PFP. This study can provide a most suitable water and N regulation model for guiding mung bean cultivation in the Mollisols region of Northeast China.

## 1. Introduction

Mung bean (*Vigna radiata* L.) is an important legume of Asian origin, now widely cultivated throughout Asia, Australia, New Zealand and Africa [1,2,3]. In China, mung beans, the main type of food legumes, are mainly planted in Northeast China and the Huang-Huai-Hai Plain. The total yield and export volume of mung beans in China ranks first in the world, with exports of about 150,000–250,000 tons. In addition, the planting area under mung bean cultivation in China has exceeded 48.5 × 10^4^ hm^2^ [4,5,6]. During the production of crops such as mung bean, corn, and wheat, the excessive N fertilizer has led to a series of environmental problems, such as air pollution, greenhouse emissions and soil acidification, which results in low N partial factor productivity (PFP) [7,8,9]. Furthermore, the yield of mung bean is restricted by irrigation water management. Unreasonable irrigation leads to an average water use efficiency (WUE) of merely 0.53 kg/m^3^ in Northeast China, whereas optimizing irrigation water volumes can achieve a WUE of 0.77 kg/m^3^ [10,11]. Therefore, an integrated consideration of the synergistic effects of irrigation and N fertilization is key to achieving sustainable mung bean production and optimizing regional water and nitrogen resources.

Optimizing the inputs of irrigation and N fertilizer is an effective way to balance a high yield of mung bean with low resource consumption [12], depending on various factors such as irrigation and fertilization methods [13]. Zhu et al. [14] found that under irrigation with upper and lower limits of 60–80%, soil water content when combined with a N application of 70 kg/ha significantly increased dry matter accumulation and the yield of mung bean compared to with no N fertilizer and irrigation. Sosiawan et al. [15] showed that drip irrigation improved the WUE of mung bean and reduced the risk of yield loss when water consumption was maintained at 80% of full irrigation, that is, 320 mm. The mung bean could make the deficit up by absorbing moisture from the deeper soil layer and groundwater through deeply extended roots [16]. However, at lower irrigation levels, excessive N fertilizer application resulted in reductions in the mung bean yield [17]. Therefore, an efficient irrigation system requires the selection of appropriate methods for crop growth, considering the coupled effects of water and N [18].

Currently, few studies have investigated the synergistic effects of optimizing water–nitrogen coupling for mung beans. However, some research indicated that water–nitrogen coupling can effectively save irrigation water and reduce N fertilizer losses, improve crop yields, dry matter accumulation, WUE and NPFP [19]. Song et al. [20] found that maintaining a single irrigation at 135 mm compared to 180 mm significantly improved water and N regulation, increased crop WUE by 1.26 times, and saved 25% of irrigation water consumption. Wang et al. [21] reported that irrigation and N application showed a coupling effect on grain WUE and NPFP when maintaining an irrigation at 140 mm. Previous studies have determined the optimal irrigation and N application based on the results of field experiments, with few inter-annual differences. However, Ballester et al. [22] optimized water and N regulation by increasing the irrigation frequency and showed that irrigation frequency and N application did not significantly affect crop dry matter accumulation and N bias productivity, in addition to significant two-year yield differences due to changing seasonal conditions. Therefore, further research on the coupling effects of optimizing irrigation and N application is necessary to elucidate these critical aspects [23].

In multi-year trials, climate, types, and soil fertilizer residual content may lead to differences in the experimental results for inter-annual trials [24]; for example, Shi [25] found that differences in rainfall caused by changes in the climate affected the stability of crop yields. However, in regions where rainfall has been negligible for three consecutive years, Behera et al. [26] simulated crop cultivation over three years using the DSSAT model and found that the yield responses as influenced by different irrigation schedules and different levels of fertilizer application were characterized by the grain yield and straw yield. Sepaskhah et al. [27] used logistic equations to quantify the influence of seasonal water and N application on maize biomass accumulation and grain yield and to develop empirical models for the prediction of maize biomass and yield. However, continuous legumes cropping did not lead to sustained improvements in soil health [28]. To characterize the comprehensive effects of continuous cropping, Gao et al. [29] established a multi-objective optimization model based on genetic algorithms to determine the amount of irrigation water and N application suitable for spring wheat cultivation in semi-arid regions of China. Similar to genetic algorithms, the TOPSIS method is used to compare multiple metrics to select the optimal proposal for aiding farmers and decision makers in developing strategies for effective inputs of water and N application, which can reduce the bias caused by subjective factors [30]. Therefore, this paper used TOPSIS to optimize water and nitrogen management.

The Northeast Plain covers a total area of about 1.03 million hectares and has now become one of the world’s major commercial grain production bases [31]. Mollisols in Northeast China have high organic matter contents and are the most fertile soils in China, with physical and chemical properties suitable for planting various crops [32]. However, the unreasonable water–N coupling model leads to a decrease in soil fertility in the Mollisols region. Excessive fertilizer input has caused a series of problems, such as low WUE and NPFP, which directly affect the stability of regional grain yield [33]. Drip irrigation coupled with fertigation can significantly improve crop yield and the efficiency of water and N coupling in arid and semi-arid zones, and improve problems caused by irrational irrigation and N applications [34,35]. Farmers grow mung beans with the expectation that high yields and quality can be achieved while saving irrigation water and N application. Therefore, this study aims (1) to investigate the effects of water and N regulation on mung bean growth, yield, dry matter, water use efficiency and N fertilizer biased productivity; and (2) to determine the optimal irrigation water volume and N fertilizer application rate for high-yield drip-irrigated mung bean production using the TOPSIS method. The results of this study will provide a theoretical basis and scientific guidance for achieving high yields and reducing water and N application to develop sustainable and friendly agriculture in drip irrigation mung bean cultivation in the Mollisols region of Northeast China.

## 2. Results

### 2.1. Agronomic Trait

**Figure 1:**
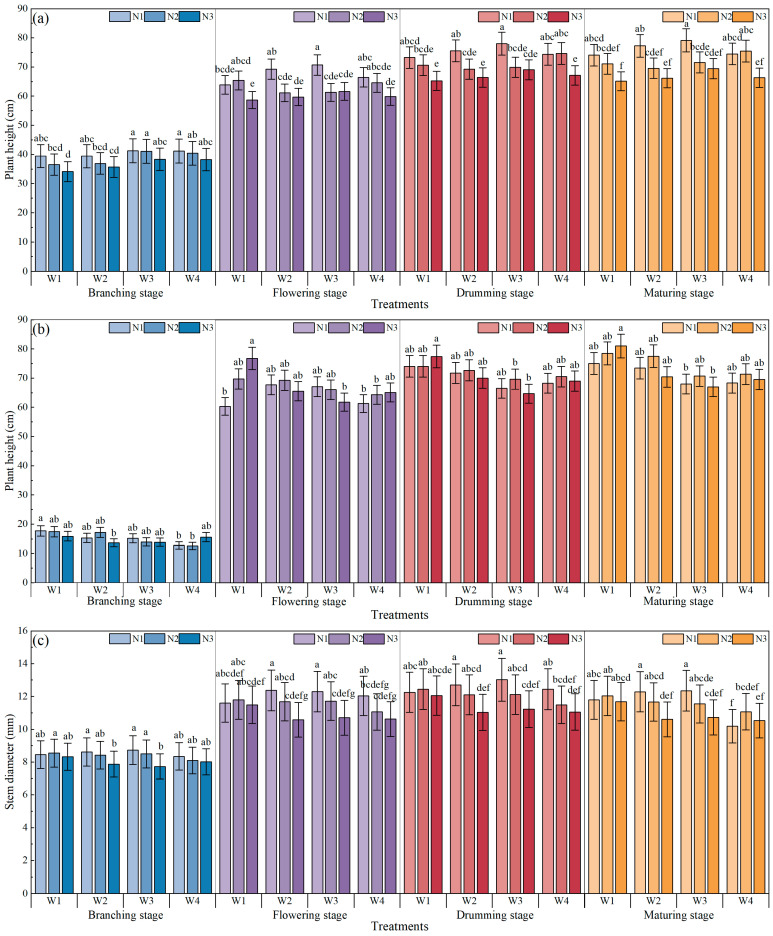

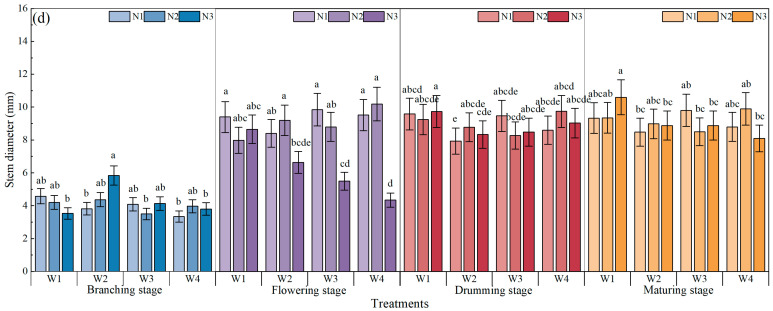
The effects of water and N regulation on plant height in (**a**) 2021 and (**b**) 2022. The effects of water and N regulation on plant stem diameter in (**c**) 2021 and (**d**) 2022. Note: Different lowercase letters represent different levels of significance for the same phenological stage within different irrigation and N fertilization treatments at *p* ≤ 0.05. W represents irrigation level. W1, 80~100% of field capacity; W2, 70~90% of field capacity; W3, 60~80% of field capacity; W4, rainfed condition. N represents nitrogen application rate level. N1, 40 kg/ha; N2, 80 kg/ha; N3, 120 kg/ha.

Figure 1 shows the effects of water and N regulation on the plant height and stem diameter of mung bean. In both 2021 and 2022, the plant height of mung bean increased the most from branching to flowering and gradually stabilized during the drumming stage. In 2021, under the same irrigation conditions, the plant height showed a trend of decreasing with increasing N applications. Under the N1, the plant height increased consistently from W1 to W3, then decreased. Under the N2 and N3, plant height first decreased and then increased with decreasing irrigation. Overall, the W3N1 treatment with respect to plant height showed an increase of 19.4% compared with W4N1. Moreover, the plant height was the highest under the W3N1 treatment. In 2022, under W1, the plant height increased with increasing N applications except at the branching stage. The W1N3 had the highest plant height, which was 26.19% higher than W4N1.

In 2021, under the W2, W3, and W4, the stem diameter showed a decreasing trend with increasing N applications. Under the same irrigation conditions, the stem diameter in the W1 treatment showed an increasing trend, followed by a decreasing trend with increasing N applications. The stem diameter under the W3N1 treatment was the highest, which was 4.63%, 2.19%, 4.61% and 21.26% higher than each of the W4N1 treatments at the branching, flowering, drumming, and maturing stages, respectively. In 2022, under the same N application conditions, the stem diameter in N1 treatments showed a decreasing–increasing–decreasing trend with decreasing irrigation. The stem diameter under the W1N3 treatment was the highest at the drumming and maturing stages with an average increase of 7.75% and 30.86% compared to W4N3.

### 2.2. Aboveground Dry Matter

**Figure 2:**
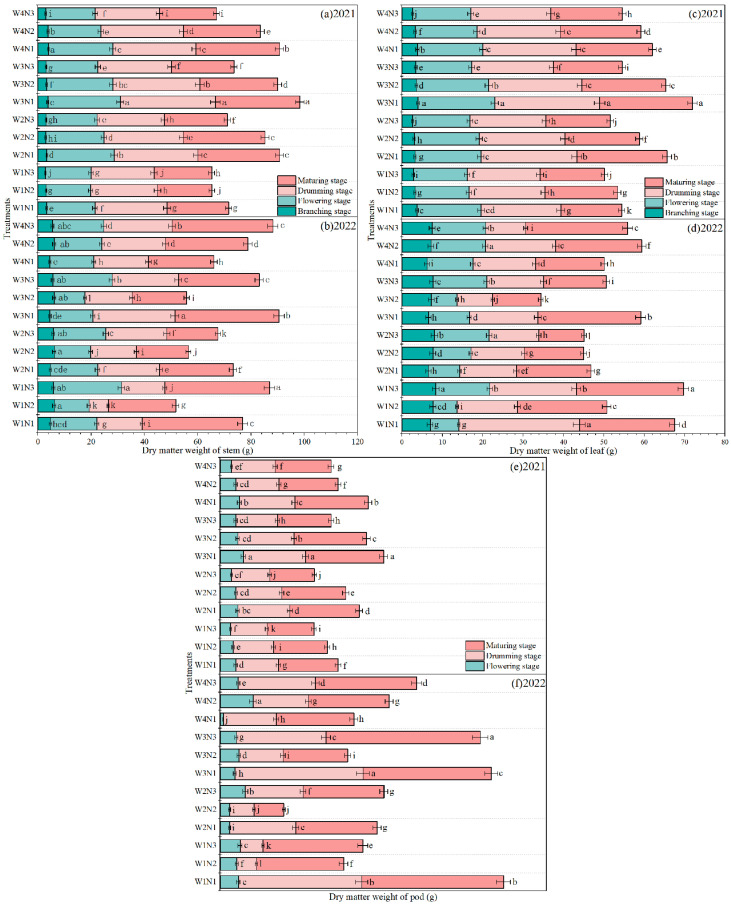
The effects of water and N regulation on mung bean aboveground dry matter of stem at the branching stage, the flowering stage, the drumming stage and the maturing stage in (**a**) 2021 and (**b**) 2022. The effects of water and N regulation on mung bean aboveground dry matter of leaf at the branching stage, the flowering stage, the drumming stage and the maturing stage in (**c**) 2021 and (**d**) 2022. The effects of water and N regulation on mung bean aboveground dry matter of pod at the branching stage, the flowering stage, the drumming stage and the maturing stage in (**e**) 2021 and (**f**) 2022. Note: Different lowercase letters represent different levels of significance for the same phenological stage within different irrigation and N fertilization treatments at *p* ≤ 0.05. W represents irrigation level. W1, 80~100% of field capacity; W2, 70~90% of field capacity; W3, 60~80% of field capacity; W4, rainfed condition. N represents nitrogen application rate level. N1, 40 kg/ha; N2, 80 kg/ha; N3, 120 kg/ha.

The effects of water and N regulations on mung bean aboveground dry matter are shown in Figure 2. In 2021, under the same irrigation conditions, the aboveground dry matter showed a decreasing trend with increasing N applications. Under the same condition of N application, the aboveground dry matter showed an increasing trend followed by a decreasing trend with decreasing irrigation except at the branching stage, and reached a maximum at W3. Compared with N3, N1 and N2 increased the aboveground dry matter by 13.47–32.36% and 6.53–19.79% at the drumming stage and by 7.32–38.61% and 4.88–28.51% at the maturing stage in 2021, respectively. Under the same N application conditions, the leaf, stem and pod of all treatments showed an increasing trend followed by a decreasing trend with decreasing irrigation, and reached a maximum at the drumming stage. Under the same irrigation conditions, the leaf, stem and pod decreased with increasing N applications. The aboveground dry matter under the W3N1 treatment was the highest at the flowering, drumming and maturing stages, with an increase of 15.1%, 13.1% and 8.36% compared with the W4N1 treatment. In 2022, the pod of the W3N1 treatment increased by 27.83 g from the flowering to drumming stages, which was the highest among all treatments. The highest aboveground dry matter was observed under the W3N1 treatment, with an increase of 1.84%, 61.68% and 58.49% compared with W4N1.

### 2.3. Yield and Yield Components

**Figure 3:**
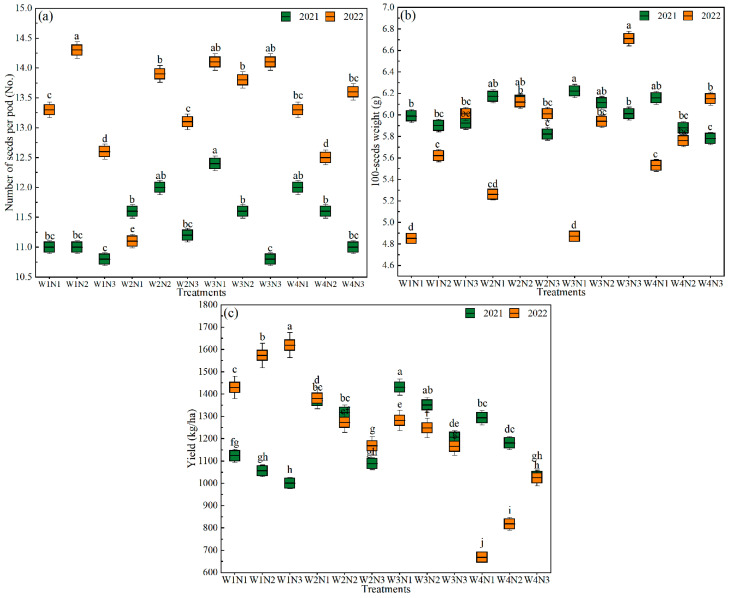
The effects of water and N regulation on the number of seeds per pod of mung bean in (**a**) 2021 and 2022, the 100-seeds weight of mung bean in (**b**) 2021 and 2022, and the yield of mung bean in (**c**) 2021 and 2022. Note: Different lowercase letters represent different levels of significance within different irrigation and N fertilization treatments at *p* ≤ 0.05 in 2021 and 2022, respectively. W represents irrigation level. W1, 80~100% of field capacity; W2, 70~90% of field capacity; W3, 60~80% of field capacity; W4, rainfed condition. N represents nitrogen application rate level. N1, 40 kg/ha; N2, 80 kg/ha; N3, 120 kg/ha.

The effects of the water and N regulation on the number of seeds per pod, 100-seeds weight and yield of mung bean are shown in Figure 3. The numbers of seeds per pod were higher in 2022 than in 2021 for all irrigation and N application treatments except the W2N1 treatment. In 2021, the number of seeds per pod was lower in W1N1, W1N2 and W1N3 treatments than that in the other N1 and N2 levels. Under W3 and W4, the number of seeds per pod decreased with increasing N applications. The number of seeds per pod under the W3N1 treatment was the highest at 12.4 with an increase of 3.33% compared with the W4N1 treatment. In 2022, the W1N2 treatment had the highest number of seeds per pod at 14.3, which was 14.4% higher than the W4N1 treatment.

In 2021, under the same irrigation conditions, the 100-seeds weight of mung bean showed a decreased trend with increasing N applications. The 100-seeds weight under the W3N1 treatment was the highest, which was 1.04% higher than the W4N1 treatment. In 2022, at the same irrigation conditions, the 100-seeds weight showed an increasing trend with increasing N applications. Compared with W4, the W3N3 treatment increased the 100-seeds weight by 9.11% and was the highest among all treatments.

Compared with N3, N1 and N2 increased yield by 12.23–25.86% and 5.57–21.04% in 2021, respectively. The W3N1 treatment had the highest yield among all the treatments at 1430.45 kg/ha with an increase of 10.58% compared with the W4N1 treatment. In 2022, the yield under W1 and W4 showed an increasing trend with increasing N applications, whereas that under W2 and W3 showed a decreasing trend with increasing N applications. Under the same N application conditions, the yield showed a decreased trend with decreasing irrigation. Compared with W4, W1, W2 and W3 increased the yield by 58.02–113.83%, 13.94–106.61% and 13.72–91.72% in 2022, respectively. The yield under the W1N3 treatment was the highest at 1619.45 kg/ha

### 2.4. Water Use Efficiency and N Partial Factor Productivity

**Figure 4:**
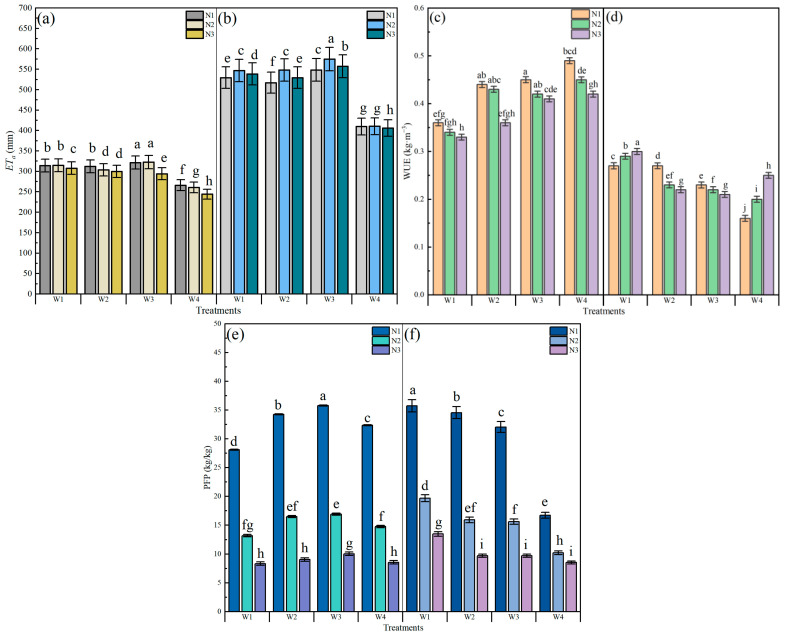
Effects of water and N regulation on actual evapontranspiration in (**a**) 2021 and (**b**) 2022, water use efficiency in (**c**) 2021 and (**d**) 2022, and N partial factor productivity in (**e**) 2021 and (**f**) 2022. Note: “ET_a_” is the actual evapontranspiration. “WUE” is the abbreviation of water use efficiency. “PFP” is the N partial factor productivity. Different lowercase letters represent different levels of significance within different irrigation and N fertilization treatments at *p* ≤ 0.05.

The effects of water and N regulation on ${\mathrm{ET}}_{a}$, WUE and NPFP are shown in Figure 4. The water consumption of W1, W2 and W3 showed a higher amount than W4 in 2021 and 2022. In 2021, under W1, W2, W3 and W4, water consumption decreased with increasing N applications. In 2022, water consumption under the N2 application level was significantly higher than N1 and N3 at the same irrigation conditions (*p* ≤ 0.05), except under W4. In 2021, the WUE and NPFP decreased with increasing N applications at the same irrigation conditions. Under the same N application levels, the WUE had an increasing trend with decreasing irrigation, whereas the PFP first increased followed by decreasing and reached a maximum at W3. The highest WUE was found in the W4N1 treatment at 0.49 kg·m^−3^. The NPFP under the W3N1 treatment was the highest at 35.76, which increased by 10.58% compared with that in the W4N1 treatment. In 2022, under W1 and W4, the WUE showed an increasing trend with increasing N applications, whereas it decreased at W2 and W3. The WUE was the highest among all treatments in the W1N3 treatment at 0.30 kg·m^−3^, which was higher than the W4N3 treatment by 20%. The differences in NPFP between treatments were consistent between the two years, i.e., the NPFP decreased with increasing N applications under the same irrigation conditions. The NPFP under the W1N1 treatment increased by 113.88% compared with the W4N1 treatment.

### 2.5. Optimization of Efficient Water and N Regulation System for Mung Bean in Mollisols Region of China on the TOPSIS Model

Table 1 shows the score rankings for each treatment and also shows that the W3N1 treatment has the highest total score ranking in 2021 and 2022, and the W2N1 treatment is ranked 2 in all treatments, just behind the W3N1 treatment. In both 2021 and 2022, the W1N3 treatment has the lowest total score ranking. Under N2 and N3, the total score rankings of different irrigation conditions are lower than the N1 application and the rankings decreased with increasing N applications. Among all treatments, only the total score rankings of treatments at the W3 irrigation condition remain constant between the two years.

When performing data calculations, the following parameters were set as target variables: the number of seeds per pod, 100-seeds weight, yield, WUE, NPFP, total water consumption, and nitrogen application rate. Among these, the total water consumption and nitrogen application rate were extremely small parameters requiring normalization. We calculated the weight of each indicator and computed its entropy value. We constructed a standardized matrix and calculated positive and negative ideal solutions, thereby obtaining relative proximity scores for ranking purposes.

## 3. Discussion

### 3.1. Effects of Different Water and N Regulations on Agronomic Traits, Aboveground Dry Matter, Yield and Yield Components

Plant height and stem diameter are important growth indicators of crop. Plant height reflects the growth of crops, and the main stem and branches are the main organs for transporting nutrients and water [36]. The synergistic effects of water and N applications affected the soil water status and soil N content where nutrients from transportation by main stem and branches were absorbed by plant roots, and thereby affected the aboveground dry matter accumulation and yield components [37,38]. In this study, the drought tolerance of mung beans increased the efficiency of soil nutrient uptake by the main stem when the lower limit of soil water content was maintained at 60% of field capacity, therefore increasing the plant height and stem diameter [39]. However, the application of large amounts of N fertilizers results in the N being unable to be fully dissolved in water and well absorbed by crops. Also, the residual N will lead to a higher N content in the soil, which could easily result in soil N leaching. In addition, the accumulation of large amounts of NO_3_^−^ ions in the soil inhibits root activity, thereby affecting crop growth [40,41]. The root depth may become shallower with increased irrigation, and most of the N is leached to deeper layers, making it difficult to be utilized by mung bean [42]. When the soil N content exceeds a reasonable range, crop growth can be negatively affected by the reduced stress tolerance and excess nutrient growth, ultimately leading to reduced dry matter accumulation and yield [43]. For mung bean, appropriate irrigation and nitrogen application rates are conducive to improving the N fixation capacity of rhizobia and increasing the effect of water–nitrogen coupling [44].

Many studies have shown that irrigation and N fertilization significantly affect crop growth, dry matter accumulation and yield, and yet the inter-annual differences in plant growth reflect the actual utilization of irrigation and N application [45]. In this study, plant height, stem diameter, aboveground dry matter accumulation and yield of mung bean decreased with increasing irrigation and N applications, reaching a maximum under the W3N1 treatment in 2021. However, at W1 in 2022, plant height, stem diameter and yield increased with increasing N applications. The reason for the inter-annual difference could be that the higher average air temperature in 2021 inhibited the photosynthesis of mung bean and led to a decrease in stomatal conductance, and these induced drought tolerance in response to low irrigation and N application [46,47]. In 2022, increasing irrigation frequency and total irrigation improved the uptake and conversion of the N fertilizer in mung bean. Furthermore, water is still the most critical factor in crop yield formation in the semi-arid region [48]. Under W3 in 2022, mung bean had the highest aboveground dry matter accumulation without the highest yield, which could be that more dry matter was transferred by the crop to the roots and stems, not to the pods, at lower soil water contents [49]. In addition, due to increased planting densities, the stem strength decreased, leading to mung bean collapse along ridges, thus inhibiting nutrient accumulation in pods during the drumming and the maturing stages [50].

The multifaceted manifestation was the reduction in crop yields due to increased temperatures, such as the number of seeds per pod in 2021 being lower than that in 2022. The average air temperature during the mung bean growth period in 2021 was 0.9 °C higher than in 2022, which may inhibit pod formation due to physiological stress [51]. Furthermore, the number of seeds per pod decreased as a result of increasing water deficit stress with increasing N applications under W3 and W4. Excessive deficit irrigation could reduce the yield threshold to meet the biomass required to acclimatize the crop [52]. Previous studies showed that fertilizer application had no significant effect on the 100-grain weight of mung bean [53]. However, as the plant grows, rhizobia gradually increase their N fixation capacity, which can contribute to the transport of N to the seed. In 2022, the highest 100-seeds weight was observed at N3 [54].

### 3.2. Effects of Different Water and N Regulations on WUE, PFP and Optimal Water and N Regulation Based on the TOPSIS

Irrigation water use efficiency is an important index to evaluate the water use of irrigation regions [55,56]. Compared with W1, W2 and W4, an appropriate increase in irrigation could improve the evapontranspiration of mung bean at W3, which indicated that mung bean has a certain degree of drought tolerance. Also, the lack of irrigation could lead to a decline in the utilization of soil nitrogen by plants, thereby affecting crop yields [57,58]. Therefore, the WUE in 2021 showed a decreasing trend with increasing irrigation volume. W3 (0.41–0.45 kg/m^3^) > W2 (0.36–0.44 kg/m^3^) > W1 (0.33–0.36 kg/m^3^). W4, with no irrigation, exhibited the highest WUE at 0.42–0.49 kg/m^3^. In 2022, crop nitrogen fixation capacity was activated, leading to increased N uptake. As nitrogen application rates increased, crop yields showed a significant improvement at W1 (*p* ≤ 0.05). The yields and WUE were highest in W1N3, reaching 1619.45 kg/ha and 0.30 kg/m^3^, respectively (Figure 3c).

N partial factor productivity is an important indicator of the combined effects of local soil-base nutrient levels and fertilizer dosage [59]. In both 2021 and 2022, PFP decreased with increasing N applications. The NPFP showed minor differences between the two years, with a PFP of 8.3–35.8 kg/kg in 2021 and 8.5–35.7 kg/kg in 2022. When excessive N fertilizer accumulated in the root zone, the plant root system may have been impaired and led to a reduction in N uptake and resulted in a decrease in PFP [60]. Zhang et al. [61]. found that an appropriate increase in irrigation under reduced N conditions facilitated the water N potential, reflecting the coupling relationship between water and N. The results of our study suggest that reasonable amounts of irrigation and N application can be utilized to achieve better results in the Mollisols region of China.

Based on TOPSIS, the present study concluded that the W3N1 (maintained 60~80% of field capacity and N application at 40 kg/ha) treatment was the most efficient treatment. In both 2021 and 2022, the W3N1 treatment had the smallest distance to the positive ideal solution and the largest distance to the negative ideal solution, with a ranking of 1 in both simulations.

## 4. Materials and Methods

### 4.1. Study Area

**Figure 5:**
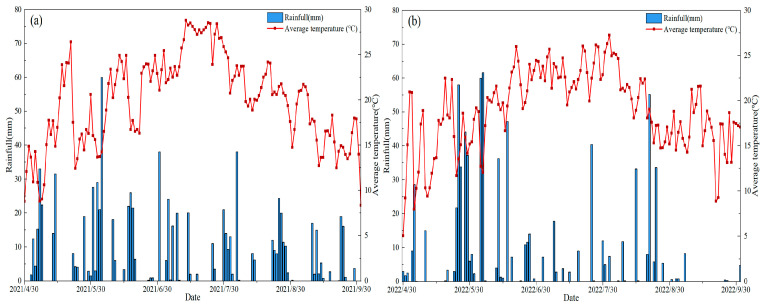
The rainfall and average temperature during the cultivation of the mung bean crop in (**a**) 2021 and (**b**) 2022.

The experiment was conducted in Xinyi village, Xiangfang District, Harbin City, Heilongjiang Province (126°44′31.60″ E, 45°44′4.65″ N, altitude 151 m), from 20 May (the sowing time) to 3 September 2021 (the harvest time), and 26 May to 6 September 2022. This area has an average annual surface evaporation of 1508 mm, an average annual precipitation of 423 mm and a frost-free period of 141 days. The climate is characterized as a temperate continental monsoon climate [61]. The average annual change in effective cumulative temperature is 2757.8 °C. The annual average temperature is 5.6 °C and the annual sunshine hours are 2600 h. According to the Department of Agriculture (USDA) soil taxonomy, the soil type for testing is Mollisols [62]. The basic properties of the experimental soil are shown in Table 2. The experiment used the potassium dichromate heating method to determine organic matter, the semi-micro Kjeldahl method to determine TN, and sodium hydroxide alkali fusion–molybdenum–antimony colorimetric method for TP, sodium hydroxide alkali fusion–flame photometry for TK, alkaline hydrolysis diffusion method for AN, molybdenum–antimony colorimetric method for AP, flame photometer for readily AK, and pH meter for pH [63]. On 18 May 2021, soil samples were collected from the middle of each plot by soil auger at 5 points in the 0–20 cm soil layer. Rainfall and average air temperature during the mung bean growing periods are shown in Figure 5. The rainfall and average temperature during the cultivation of the mung bean for 2021 and 2022 were 407 mm and 21.4 °C and 406.7 mm and 20.5 °C, respectively.

### 4.2. Experimental Design

The field experiment was laid out in a split-plot design. Four levels of soil water content and three N application treatments were designed. The irrigation treatments were as follows: W1, 80~100% of field capacity; W2, 70~90% of field capacity; W3, 60~80% of field capacity; W4, rainfed condition, no irrigation. The N application treatments were as follows: N1, 40 kg/ha; N2, 80 kg/ha; N3, 120 kg/ha. The experimental treatment design is shown in Table 3.

Each treatment was repeated 3 times, with a total of 36 experimental plots, each with an area of 22.75 m^2^ (3.25 m × 7 m), and was surrounded by protection rows, with the width of the protective rows between the plots being 1 m. The tested variety was “White Green 522”. The seeds were selected and then sun-dried for 1–2 d to improve the vitality of mung bean seeds. Sowing was started when the ground temperature stabilized at 12 °C at a depth of 5 cm of soil and maintained in 30 cm apart rows. A total of 15 days after sowing, mung bean plants were thinned out to keep the spacing between plants at about 6–7 cm. The amount of potassium and phosphate fertilizer applied was consistent across the plots of the experiment. Calcium superphosphate (12% P_2_O_5_, 75 kg/ha) and potassium sulfate (50% K_2_O, 150 kg/ha) were selected as the basal applications. One-third of the urea (N, 46%) was applied with the seed fertilizer, and the remaining N fertilizer was applied using fertigation at the beginning of the mung bean’s flowering stage. N fertilizer was applied using fertigation. Drip irrigation was used in the experiment and the drip irrigation belts were laid at the root position of mung bean with a flow rate of 1.5 L/h. Each plot was equipped with an independent branch control unit, including a water meter, gate valve, and pressure gauge. Soil water content was measured every 7 days with a soil auger. Irrigation occurred when the soil water content reached the lower limit of irrigation.

The experiment sampling started at the branching stage while manual weeding was used to avoid weeds harming the growth and development of mung bean. Observations and records were made at the branching stage, the flowering stage, the drumming stage, the maturing stage and the picking stage of mung bean.

### 4.3. Data Collection

#### 4.3.1. Mung Bean Growth

Ten plants were selected in each plot to represent the overall growth in the plot and each plant was marked, and the stem diameter and plant height were measured and averaged. Stem diameter was measured using Vernier calipers with an accuracy of 0.1 mm. Plant height was measured using a steel ruler with an accuracy of 1 mm.

#### 4.3.2. Yield and Yield Components

At maturity, mung beans from all treatments in a 3.25 m × 5 m area were harvested and weighed on an electronic balance (0.01 g) and measured for 100-seeds weight and number of seeds per pod from the 10 mung bean plants marked in each plot. The yield of each plot was calculated, and the total yield of mung bean per hectare was determined for the different treatments according to the planting density.

#### 4.3.3. Aboveground Dry Matter

Six mung bean plants with representative growth were selected at the end of each growth stage. The stems, leaves and pods of mung bean were collected and placed into different paper bags. Separate organs were placed in an oven set at 105 °C for 30 min, followed by drying at 80 °C until constant weight and aboveground dry matter was weighed on an electronic balance (0.01 g).

#### 4.3.4. N Fertilizer Partial Factor Productivity

The N partial factor productivity (PFP) was calculated according to the following formula [64]:

(1) PFP=Y/FT

where *PFP* is the partial factor productivity, *Y* is the mung bean yield (kg/ha), and *FT* is the total N fertilizer application (kg/ha).

#### 4.3.5. Water Consumption and Water Use Efficiency

Irrigation was carried out when soil water content was below the lower limit of soil water content. Irrigation amount was calculated according to the following formula [65]:

(2) $$I=r\left(\theta_{u}-\theta \right)H/10$$

where *I* is irrigation water quota (mm); r is volume mass (g/cm^3^); H is planned wetting depth (0–60 cm soil layer, cm); $\theta_{u}$ is the upper limit of soil water content (%); θ is soil water content of planned wetting depth (0–60 cm) before irrigation (%).

Soil water content in the 0–60 cm soil layer of each plot was measured at the beginning and end of each growth stage. Soil samples were collected at each plot using a soil auger, and soil water content was measured using the oven-drying method [66]. Crop actual evapotranspiration (*ET_a_*) was calculated using the water balance equation:

(3) $${ET}_{a}=W_{1}+I+J-W_{2}$$

where ${ET}_{a}$ is the actual water consumption by the mung bean during the branching stage, flowering stage, drumming stage and maturing stage (mm); $W_{1}$ is the soil water content at the beginning of the growth stage (mm); I is the irrigation water quota (mm); J is the amount of rainfall (mm); $W_{2}$ is the soil water content at the end of each growth stage (mm).

The water use efficiency (WUE) of each plot was calculated [67] by

(4) $$WUE=\frac{Y}{{ET}_{a}\times 10}$$

where *WUE* is the water use efficiency (kg·m^−3^); Y is the yield (kg/ha); ${ET}_{a}$ is the actual evapotranspiration by the crop during the period (mm).

#### 4.3.6. Multi-Objective Decision Making and TOPSIS Evaluation

The technique for order preference by similarity to ideal solution (TOPSIS) was used to identify a solution from the feasible solution set by defining the positive ideal solution and the negative ideal solution for the decision problem so that the feasible solution was closest to the positive ideal solution and farthest from the negative ideal solution [68].

Twelve treatments were set as evaluation objects, with eight evaluation indicators including number of seeds per pod, 100-seeds weight, yield, ${\mathrm{ET}}_{a}$, FT, WUE and PFP. The evaluation indicators were normalized to establish a normalized matrix:

(5) $$Z_{ij}=\frac{x_{ij}}{\sqrt{\sum_{i=1}^{n}x_{ij}^{2}}}$$

where $Z_{ij}$ is the *j* index normalized value in *i* treatment; $x_{ij}$ is the *j* index value in the *i* treatment. *I* = 1, 2, …, n; j = 1, 2, …, m.

The ideal solution ($Z_{ij}^{+}$) and the negative solution ($Z_{ij}^{-}$) were determined to form the ideal solution vector $Z^{+}$ and the negative solution vector $Z^{-}$, respectively:

(6) $$Z_{ij}^{+}=\left(z_{i1}^{+},\;z_{i2}^{+},\;z_{i3}^{+}\;\ldots \ldots z_{ij}^{+}\right)$$

(7) $$Z_{ij}^{-}=\left(z_{i1}^{-},\;z_{i2}^{-},\;z_{i3}^{-}\;\ldots \ldots z_{ij}^{-}\right)$$

where $Z_{ij}^{+}$ and $Z_{ij}^{-}$ represent the maximum and minimum values of the evaluation object in the *j*-th index, respectively.

The Euclidean distances ($D_{i}^{+}$ and $D_{i}^{-}$) were determined:

(8) $$D_{i}^{+}=\sqrt{\sum_{j=1}^{m}{\left[w_{j}\times \left(z_{ij}-Z_{ij}^{+}\right)\right]}^{2}}$$

(9) $$D_{i}^{+}=\sqrt{\sum_{j=1}^{m}{\left[w_{j}\times \left(z_{ij}-Z_{ij}^{+}\right)\right]}^{2}}$$

where $w_{j}$ is the weight of indicator *j*.

The relative proximity coefficient $C_{i}$ of each treatment was calculated; that is, the proximity between the evaluation object and the optimal scheme was calculated as follows:

(10) $$C_{i}=\frac{D_{i}^{-}}{D_{i}^{+}+D_{i}^{-}}$$

### 4.4. Statistical Analysis

Significant effects of treatments were determined by analysis of variance (ANOVA) using Minitap19 (Minitab Inc., State College, PA, USA), and treatments were compared by the Turkey’s Honest Significant Difference (Turkey’s HSD) test. All statistical analyses in this paper were performed by Minitap19 and plotted with Origin 2023 (Origin Lab corporation, Northampton, MA, USA). Microsoft Excel 2016 (Microsoft Corp., Redmond, WA, USA) was used for data collation.

## 5. Conclusions

This two-year field study evaluated the combined effects of irrigation regimes and nitrogen application rates on mung bean production in the Mollisols region of Northeast China under drip irrigation. The results demonstrated that excessive irrigation and nitrogen input did not consistently improve crop growth or yield, but instead reduced water and nitrogen use efficiencies. The W3N1 treatment had a higher ranking than the other treatments based on TOPSIS. Maintaining the soil water content at 60~80% of the field capacity and applying N at 40 kg/ha achieved optimized irrigation water and nitrogen application rates and ensured the sustainable production of multi-year mung bean in the Mollisols region of Northeast China.

## Figures and Tables

**Table 1:** Evaluation results of calculation of TOPSIS.

Year	Treatments	$D_{i}^{+}$	$D_{i}^{-}$	$C_{i}$	Rank	Year	Treatments	$D_{i}^{+}$	$D_{i}^{-}$	$C_{i}$	Rank
2021	W1N1	0.069	0.107	0.606	4	2022	W1N1	0.098	0.099	0.504	3
	W1N2	0.137	0.027	0.165	10		W1N2	0.162	0.034	0.173	11
	W1N3	0.163	0.003	0.019	12		W1N3	0.189	0.003	0.017	12
	W2N1	0.021	0.149	0.878	2		W2N1	0.070	0.148	0.677	2
	W2N2	0.105	0.067	0.391	6		W2N2	0.140	0.065	0.316	7
	W2N3	0.153	0.017	0.099	11		W2N3	0.173	0.065	0.273	9
	W3N1	0.013	0.159	0.924	1		W3N1	0.039	0.178	0.822	1
	W3N2	0.104	0.068	0.395	5		W3N2	0.121	0.081	0.402	5
	W3N3	0.142	0.037	0.205	8		W3N3	0.163	0.073	0.308	8
	W4N1	0.024	0.142	0.856	3		W4N1	0.146	0.131	0.472	4
	W4N2	0.116	0.058	0.332	7		W4N2	0.176	0.101	0.364	6
	W4N3	0.154	0.031	0.170	9		W4N3	0.187	0.069	0.269	10

Note: “$D_{i}^{+}$” and “$D_{i}^{-}$” are the Euclidean distances, “$C_{i}$” is the relative closeness.

**Table 2:** Properties of the tested soil.

Properties	Organic Matter (g/kg)	TN (g/kg)	TP (g/kg)	TK (g/kg)	AN (mg/kg)	AP (mg/kg)	AK (mg/kg)	pH Value
Value	27.41	1.01	0.77	21.30	110.42	45.98	196.51	7.1

Note: “TN” is the abbreviation of “total N”. “TP” is the abbreviation of “total phosphorus”. “TK” is the abbreviation of “total potassium”. “AN” is the abbreviation of “alkaline N”. “AP” is the abbreviation of “available phosphorus”. “AK” is the abbreviation of “available potassium”.

**Table 3:** Design of experimental treatments.

No.	Treatments	Irrigation Mode	N Application (kg/ha)	Total Amount of Water (mm)	
				2021	2022
1	W1N1	80~100% ɸ	40	314.45	529.49
2	W1N2	80~100% ɸ	80	314.89	546.65
3	W1N3	80~100% ɸ	120	307.85	538.64
4	W2N1	70~90% ɸ	40	312.3	517.16
5	W2N2	70~90% ɸ	80	303.6	548.15
6	W2N3	70~90% ɸ	120	299.62	529.40
7	W3N1	60~80% ɸ	40	321.39	548.42
8	W3N2	60~80% ɸ	80	322.46	575.05
9	W3N3	60~80% ɸ	120	294.02	557.12
10	W4N1	Rainfed	40	266.17	409.64
11	W4N2	Rainfed	80	260.62	410.59
12	W4N3	Rainfed	120	244.06	406.22

Note: “ɸ” is the field capacity. “Rainfed” indicates no irrigation.

## Data Availability

Data are contained within the article.
